# RNA Helicase DDX6 Regulates A-to-I Editing and Neuronal Differentiation in Human Cells

**DOI:** 10.3390/ijms24043197

**Published:** 2023-02-06

**Authors:** Chia-Yu Shih, Yun-Chi Chen, Heng-Yi Lin, Chia-Ying Chu

**Affiliations:** 1 Department of Life Science, College of Life Science, National Taiwan University, Taipei 10617, Taiwan; 2 Center for Systems Biology, National Taiwan University, Taipei 10617, Taiwan

**Keywords:** RNA editing, RNA modification, RNA helicase, DDX6, A-to-I editing, ADAR, neuronal differentiation

## Abstract

The DEAD-box proteins, one family of RNA-binding proteins (RBPs), participate in post-transcriptional regulation of gene expression with multiple aspects. Among them, DDX6 is an essential component of the cytoplasmic RNA processing body (P-body) and is involved in translational repression, miRNA-meditated gene silencing, and RNA decay. In addition to the cytoplasmic function, DDX6 is also present in the nucleus, but the nuclear function remains unknown. To decipher the potential role of DDX6 in the nucleus, we performed mass spectrometry analysis of immunoprecipitated DDX6 from a HeLa nuclear extract. We found that adenosine deaminases that act on RNA 1 (ADAR1) interact with DDX6 in the nucleus. Utilizing our newly developed dual-fluorescence reporter assay, we elucidated the DDX6 function as negative regulators in cellular ADAR1p110 and ADAR2. In addition, depletion of DDX6 and ADARs results in the opposite effect on facilitation of RA-induced differentiation of neuronal lineage cells. Our data suggest the impact of DDX6 in regulation of the cellular RNA editing level, thus contributing to differentiation in the neuronal cell model.

## 1. Introduction

Gene expression in eukaryotic cells is controlled at multiple levels. Among them, the post-transcriptional stage is critical for various mRNA processes with sophisticated regulatory mechanisms. Diverse RNA binding proteins (RBPs) are involved in many aspects of post-transcriptional regulation [1]. One group of RBPs, the RNA helicases, participate in nearly all aspects of RNA metabolism, including pre-mRNA splicing, nuclear mRNA export, RNA storage, and RNA turnover [2]. The DEAD-box proteins are the most prominent RNA helicase family with the special Asp-Glu-Ala-Asp (DEAD) amino acid sequence on motif II and the ATP-dependent RNA unwinding activity [3,4]. Many cellular processes have been shown to require the presence of DEAD-box proteins [5,6,7,8,9]. 

DDX6, one of the DEAD-box helicases, is highly conserved in eukaryotes with homologs in *Saccharomyces cerevisiae* (Dhh1P), *Caenorhabditis elegans* (CGH-1), *Drosophila* (Me31B), and *Xenopus* (Xp54). It contains two RecA-like domains with nine conserved motifs, including the unique DEAD-box and SAT motifs that might be required for its helicase activities [10,11]. The RNA-binding ability and RNA helicase activity of DDX6 contribute to its function in regulation of various pathways in RNA metabolism: processing body (P-body) formation, miRNA-mediated gene silencing, RNA decay, and translational repression [12,13,14,15]. Its conserved functions and critical roles in multiple biological processes of DDX6 homologs have been studied in various species [10,16,17,18,19]. In *Xenopus* oocytes, the p54 protein (Xp54) functions as an essential component of messenger ribonucleoprotein (mRNP) particles and participates in translational control of maternal mRNA [20]. In *C. elegans*, CGH-1 localizes to P-granules in germ cells for protection and sequestration of specific maternal mRNAs during oogenesis [21]. In somatic cells of *C. elegans*, CGH-1 forms Patr1-dependent P-bodies that are involved in mRNA decapping. In *Drosophila*, the DDX6 homolog Me31b was identified as a component of germ granules in oocytes and nurse cells. In *Drosophila* egg chambers, Me31B mediates the translational silencing of the *osk* and *Bicaudal-D* (*BicD*) mRNAs, which are critical for embryonic development [19]. 

Human DDX6 was initially identified in B-cell lymphoma, RC-K8, as a 54-kDa protein translated from a gene located at a breakpoint of chromosome 11. Overexpression of DDX6 in colorectal cancers suggested its putative function as a proto-oncogene. Significantly increased expression of DDX6 was also found in other cancer cells [22,23,24]. In gastric cancer, DDX6 positively regulated the expression of HER2 and FGFR2 and promoted the process of c-Myc [23,25]. Many studies have also revealed the multiple roles of DDX6 in facilitating translational repression in the host–pathogen response. It has been shown that DDX6 is involved in repression of HIV-1 virus replication via miR-29a in host cells [26]. In addition, the contribution of DDX6 to efficient foamy-virus genome packaging suggested the unique role of DDX6 in relocation to the site of viral assembly; thus, DDX6 may function as a catalyst in the encapsidation of retrovirus [27]. 

In addition to the functional consequences of DDX6 in cancer and viral infection, DDX6 also plays multiple roles in facilitating translational repression in cell differentiation and embryogenesis [28]. Studies on the reticulocyte 15-lipoxygenase (r15-LOX), which functions in the late stages of erythrocyte maturation from reticulocytes, have shown that DDX6 interacts with hnRNP K/E1 to bind the 3′UTR of r15-LOX mRNA and maintain the translational silencing of r15-LOX mRNA in premature erythroid cells during the maturation process of erythrocytes [29]. As a translational regulator, DDX6 was reported to coordinate mammalian epidermal progenitor cells through degradation of differentiation-inducing transcripts and trigger translation of self-renewal-related mRNA [30]. Moreover, DDX6 affects the exit of stem cells from the pluripotent state in a context-dependent manner. Specifically, it inhibits self-renewal in ectodermal progenitor cells and drives them into a differentiated state [31]. While the DDX6 function in neuronal development has not been comprehensively addressed, a recent study showed that missense variants of DDX6 exon11 could cause intellectual disability and dysmorphic features [32]. Patients with this DDX6 mutation exhibited a developmental disability with hypoplastic posterior corpus callosum and speech delay, which suggests that DDX6 may have a prominent role in neuronal development and cell differentiation. Thus, DDX6 may regulate the expression of crucial genes in the cell fate transition of neuronal differentiation. 

Although most documented cellular functions of DDX6, e.g., facilitation of translational repression or mRNA decay, are mainly located in the cytoplasm, evidence from various model organisms indicates that DDX6 homologs enter the nucleus [33,34,35,36,37]. We previously proved that the nucleocytoplasmic localization of human DDX6 is determined with dual mechanisms; the C-terminal domain of DDX6 facilitates its nuclear entry [37]. However, the function of DDX6 in nuclei beyond its role in cytoplasmic mRNA silencing remains unknown. To decrypt the role of DDX6 in the nuclei of human cells, we conducted a mass spectrometry (MS) analysis of the anti-DDX6 immunoprecipitation in nuclear extracts to identify DDX6 interactors in this study. We found that two ADAR proteins catalyze A-to-I editing of RNA in human cells and are associated with DDX6. ADAR1p110 and ADAR2 are constitutively expressed and primarily presented in the nucleus. Both ADARs target specific adenosines on double-stranded RNA and convert adenosine (A) to inosine (I) through deamination. Through inosine (I) base pairs with cytidine (C), the A-to-I editing of RNA results in changed codon recognitions, alteration of the miRNA-binding site, and post-transcriptional regulation of protein expression [38,39]. Thus, DDX6 may participate in the A-to-I modification mechanism, which is novel for the function of DDX6 in human cells.

To decipher the regulatory role of DDX6 in ADAR-mediated A-to-I RNA editing, we developed a dual-fluorescence assay to monitor ADAR activity in vivo and in vitro. This study revealed that DDX6 exerts potent effects on regulating ADAR editing activity, while the DEAD helicase motif is dispensable. Furthermore, we showed that DDX6 and ADARs participate in RA-induced neuronal differentiation of human SH-SY5Y cells, indicating the potential consequences of DDX6 on regulation of RNA editing in neuronal development.

## 2. Results

### 2.1. DDX6 Interacts with ADAR1 and ADAR2 in the Nuclei of Human Cells

**Figure 1:**
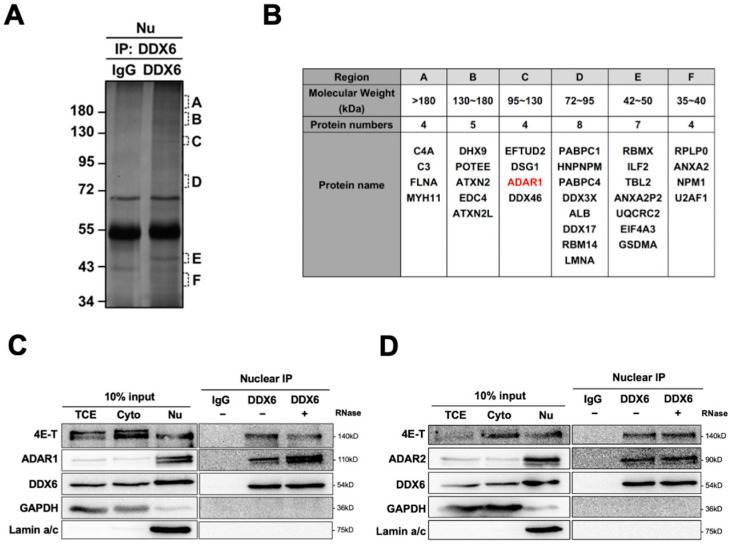
DDX6 interacts with ADARs in the nuclei of human cells. (**A**) Six regions from the silver-stained gel of an anti-DDX6 IP sample were subjected for MS analysis. Regions containing extra protein bands from the anti-DDX6 IP product are marked in alphabetical order according to their molecular weight (A: >180 kDa; B: 130–180 kDa; C: 120–130 kDa; D: 72–95 kDa; E: 42–50 kDa; F: 35–40 kDa). (**B**) A total of 36 proteins were identified in a co-IP/MS analysis from samples in regions with various molecular weights. ADAR1 was presented in red. (**C**,**D**) Anti-DDX6 immunoprecipitation was conducted in HEK293T cells that overexpressed ADAR1 (**C**) or ADAR2 (**D**), treated with +/− RNase A, and was followed by WB analysis using ADAR1, ADAR2, and DDX6 antibodies. Cytoplasmic and nuclear extracts from HEK293T cells were prepared with the Gagnon–Li method. Ten percent of the total cell lysate was used as the input control. TCE, total cell lysates; Nu, nuclear fraction; Cyto, cytoplasmic fraction; IgG, immunoglobin G; 4E-T, positive control for DDX6-IP; GAPDH, cytoplasmic marker; Lamin a/c, nuclear marker.

To identify DDX6-interacting proteins and to illuminate the potential functions of DDX6 in the nucleus, we performed a mass spectrometry analysis for immunoprecipitation of anti-DDX6 in the nuclear extracts of human cells (Figure 1A). A total of 36 proteins were identified in a Mass-Spec analysis of samples in regions with various molecular weights (Figure 1B) (Table S4). We found that human ADAR1 is present as the interactor of DDX6 in the nuclear extracts of human cells. To confirm this result, we performed immunoblotting and examined the presence of ADAR1 in anti-DDX6-coimmunoprecipitation. As shown in Figure 1C, ADAR1 was co-IPed with DDX6 in the nuclear extracts of HEK293T cells. While human ADAR2 was not detected in our MS analysis, we observed that ADAR2 also co-IPed with DDX6 in the nucleus (Figure 1D). Analysis of immunoprecipitated complexes showed that ADAR1 and ADAR2 interactions with DDX6 were not affected by RNase treatment, suggesting that DDX6 directly interacted with ADAR1 and ADAR2 and is potentially involved in regulating the function of ADARs in nuclei.

### 2.2. The Dual-Fluorescence Reporter System Is Sensitive to the RNA-Editing Activity of ADARs in Mammalian Cells

**Figure 2:**
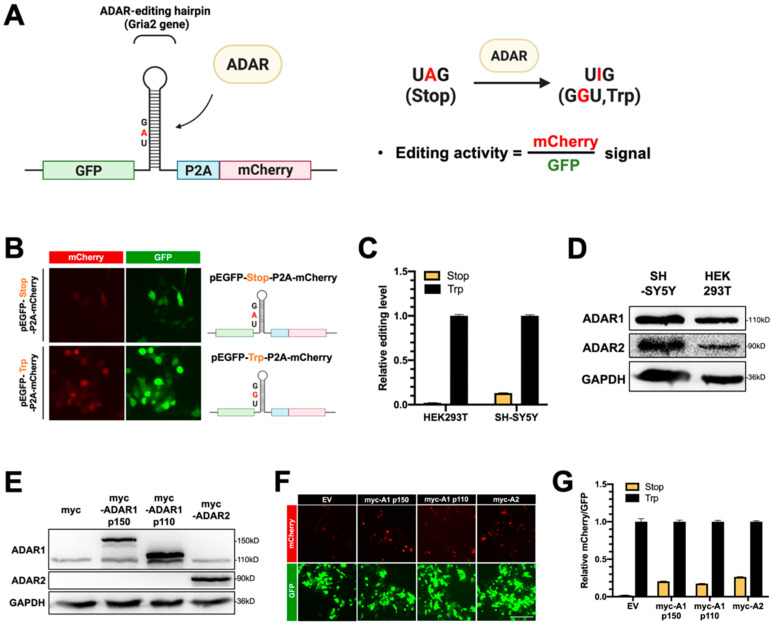
The dual-fluorescence reporter (DFR) assay is sensitive to monitoring of A-to-I editing in human cells. (**A**) Design of the DFR construct. The R/G editing site from Gria2 (GluA2) mRNA was modified as a stop codon (UAG), which would be recoded into a tryptophan codon (UGG) upon editing. The editing substrate was inserted at the linker region of the GFP/mCherry-fusion transcript. The downstream mCherry protein would be translated when the GluA2 substrate site was edited via ADARs. The ratio of mCherry/GFP signals was used to measure the A-to-I editing level. (**B**) A reporter carrying a single nucleotide mutation (A-to-G) at the editing site was used as the 100% edited control for the reporter assay (pEGFP-Trp-P2A-mCherry). Fluorescence images of HEK293T cells transfected with the two reporters are shown (left). Magnification 400×. Green, EGFP; blue, P2A; red, mCherry. (**C**) The editing activity of endogenous ADARs was lower in HEK293T cells than that in SH-SY5Y (neuroblastoma) cells. The editing level was monitored as the ratio of mCherry/GFP in pEGFP-Stop-P2A-mCherry and normalized to that in pEGFP-Trp-P2A-mCherry (the 100% edited control). Stop, pEGFP-Stop-P2A-mCherry (yellow); Trp, pEGFP-Trp-P2A-mCherry (black) (*N* = 3). (**D**) The WB result showed that both ADAR1p110 and ADAR2 were expressed in the HEK293T and SH-SY5Y cells. (**E**) WB analysis showed the expression of endogenous and exogenous ADAR1 and ADAR2 in the total cell extracts. GAPDH, the loading control. (**F**) Increased mCherry signals expressed from the reporter constructs were detected in cells transfected with various myc-ADARs. Scale bar = 500 µm. (**G**) The DFR assay showed the increased A-to-I editing level in HEK293T cells transfected with various myc-ADARs. Cells transfected with myc-empty vectors (EVs) were used as a negative control. The editing level was monitored as the ratio of mCherry/GFP in pEGFP-Stop-P2A-mCherry and normalized to that in pEGFP-Trp-P2A-mCherry (*N* = 3). Stop, pEGFP-Stop-P2A-mCherry; Trp, pEGFP-Trp-P2A-mCherry; EV, empty vector (pcDNA3.1-myc) (error bars: standard deviation).

To examine the A-to-I editing activity of ADARs and to characterize the function of DDX6 in regulation of ADARs, we developed a dual-fluorescence reporter system with inserted ADAR-editing RNA hairpin substrate between the GFP and mCherry genes in the reporter plasmid that expresses GFP-P2A-mCherry (Figure 2A,B, pEGFP-Stop-P2A-mCherry). The inserted substrate sequence was a 71 bp fragment modified from a natural editing substrate, the RNA of glutamate ionotropic receptor AMPA-type subunit 2 gene (GRIA2), with a modification to introduce a stop codon (UAG) that would be translated as a tryptophan (UIG) upon editing [40]. Thus, the mCherry gene downstream to the editing site would be expressed, and the ratio of the mCherry signal over the GFP signal could indicate the editing activity of ADARs in the cells (Figure 2A). We also generated a similar construct with a point mutation, at the editing site, to a G (UAG->UGG), which mimicked 100% editing, as the positive control in our reporter assays (Figure 2B, pEGFP-Trp-P2A-mCherry). 

To test whether this dual-fluorescence reporter system was sufficient to detect editing activity in mammalian cells, we transfected reporter constructs into HEK293T cells. We monitored mCherry and GFP signals at 48 h post-transfection. Cells transfected with pEGFP-Stop-P2A-mCherry (reporter) showed weak mCherry signals, while transfection of pEGFP-Trp-P2A-mCherry (positive control) resulted in strong signals of mCherry (Figure 2B). To quantify the mCherry and GFP signals and the editing activity of endogenous ADARs, we prepared lysates from HEK293T cells or human neuroblastoma SH-SY5Y cells transfected with reporters. Then, we measured the fluorescence in the lysates on a multiwell fluorescence reader. The editing level of reporter mRNA was measured and presented relative to the signals in cells that expressed positive control. As shown in Figure 2C, the relative editing levels of reporter mRNA catalyzed with endogenous ADARs in HEK293T cells was ~2%, while the relative editing level in SH-SY5Y was ~13% (Figure 2C). As our Western blotting data showed a higher amount of endogenous ADAR2 protein expressed in SH-SY5Y cells than in HEK293T cells (Figure 2D), we speculated that the increased ADAR editing level in the SH-SY5Y cells was the result of ADAR2-mediated A-to-I editing. Thus, we observed the differences in endogenous editing activity for these two cells with the dual-fluorescence reporter assay. 

Next, we tested whether the reporter system could detect the editing activity of exogenous ADARs. Three plasmids expressing myc-tagged ADARs, including two ADAR1 isoforms (p150 and p110) and ADAR2, were transfected into HEK293T cells for 24 h, followed by transfection of the fluorescence reporter constructs for another 24 h. All overexpressed myc-ADARs were detected in total cell extracts with Western blotting at 48 h post-transfection (Figure 2E). We observed increased mCherry signals in cells transfected with myc-ADARs (Figure 2F). We quantified the relative mCherry/GFP levels in cells transfected with myc-empty vectors or myc-tagged ADARs. As expected, the relative mCherry/GFP ratio was increased from 2% to ~20% of the positive control in cells transfected with myc-ADAR1p150. Similar effects of increased mCherry signals were observed when cells were transfected with myc-ADAR1p110 or myc-ADAR2, indicating that this reporter is sufficient to reflect ADAR editing activity (Figure 2G). Thus, our dual-fluorescence system successfully and faithfully reports ADAR activity in living cells and can be applied to examine the cellular A-to-I editing levels regulated with DDX6 and other potential factors, as in the following experiments.

### 2.3. Depletion of DDX6 Increases ADAR Editing Activity

To examine whether DDX6 regulates the function of ADAR1 and ADAR2 in human nuclei, we used the dual-fluorescence reporter system (DFR) to detect the ADAR activity in cells that overexpressed DDX6. We observed that the editing level of reporter mRNA in DDX6-overexpressed HEK293T cells was slightly decreased compared to that in control cells transfected with empty vectors, while the effects were insignificant in the statistics (Figure S1A). 

To further confirm the potential function of DDX6 as a negative regulator of ADARs, we performed the DFR assay in 293T cells transfected with siRNA against DDX6 and monitored the ADAR activity thereof. The editing levels of the reporter were slightly increased in cells treated with perfectly matched DDX6 siRNA, but the differences between the knocked-down and control samples were insignificant (Figure S1B). 

**Figure 3:**
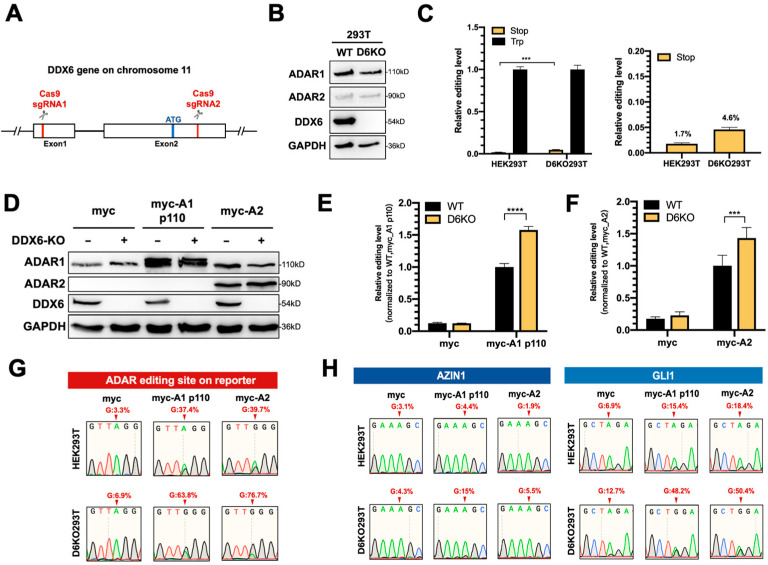
ADAR editing activity was increased in DDX6-KO HEK293T cells. (**A**) This schematic shows the CRISPR/Cas9-mediated knockout of DDX6. Two sgRNAs, targeted to exon 1 and exon 2, were cotransfected into HEK293T cells to generate DDX6 KO clones. Cas9-sgRNA induced DNA double-strand-breaks and resulted in deletion at exons 1–2. Red, sgRNA target sites; blue, start codon. (**B**) Depletion of DDX6, and no change in the expression of ADARp110 or ADAR2, were confirmed with WB analysis. (**C**) The editing level catalyzed with endogenous ADARs was increased in DDX6-KO HEK293T cells. The relative editing level was increased from 1.7% to 4.6%. Stop (yellow), pEGFP-Stop-P2A-mCherry; Trp (black), pEGFP-Trp-P2A-mCherry; (*N* = 3) (**D**) WB analysis showed the expression of myc-ADARs in cells transfected with equal amounts of plasmids. GAPDH, loading control. (**E**,**F**) The editing activity of exogenous myc-ADARs was increased in DDX6-KO HEK293T cells. The fluorescence ratio of mCherry/GFP was measured in the presence of pEGFP-Stop-P2A-mCherry and normalized to the ratio observed in the presence of pEGFP-Trp-P2A-mCherry. Black, wild-type HEK293T cells; yellow, DDX6-KO HEK293T cells. Error bars indicate the standard deviation. Statistical analysis was conducted using an unpaired, two-tailed Student’s *t* test. (**E**) *N* = 6; (**F**) *N* = 8; ***: *p* < 0.001; ****: *p* < 0.0001. (**G**) Sanger sequencing chromatograms illustrate the A-to-I editing level at the ADAR-target site (from gene GRIA2) on the reporter plasmid. The observable dual A/G peaks show the increased editing level after DDX6 KO in HEK293T cells. (**H**) Sanger sequencing chromatograms of two other genes. AZIN1 is a target of ADAR1. GLI1 can be edited via both ADAR1 and ADAR2. Arrows indicate the editing sites. Percentage of editing were determined via measurement of the relative peak height for G: (G/(A + G) × 100%).

This effect could be limited, since knockdown via RNAi could not entirely eliminate endogenous DDX6 (Figure S1B); thus, we could not observe significant changes in the editing level of the reporter. To completely remove the endogenous DDX6, we generated a DDX6-knockout HEK293T cell line (D6KO293T) using a CRISPR/Cas9 system with two sgRNAs, as illustrated in Figure 3A. Western blotting results showed elimination of DDX6 in D6KO cells (Figure 3B). Notably, the expressions of both ADAR1 and ADAR2 were not changed after DDX6 depletion. We then examined the differences in ADAR activity in DDX6-KO compared to in wild-type cells. First, we monitored the editing activity of endogenous ADARs through transfection of the DFR in WT or D6KO cells. We found that the editing level of reporter mRNA was increased in D6KO cells (Figure 3C). We next individually examined the effect of DDX6-KO on ADAR1 and ADAR2 activity. We transfected equal amounts of myc-ADAR1p110 or myc-ADAR2 in wild-type (WT) or DDX6-KO (D6KO) HEK293T cells, followed by transfection of DFR. The editing level of reporter mRNA was increased significantly in D6KO cells (Figure 3D–F), suggesting that DDX6 functions as a negative regulator of ADARs. 

To confirm that the A-to-I editing level in reporter mRNA was indeed increased upon DDX6 depletion, we PCR-amplified the ADAR target region on the cDNA of the reporter transcripts and subjected it to Sanger sequencing (Figure 3G). As expected, the dual peaks of A/G were observed in HEK293T cells transfected with myc-ADAR1 and myc-ADAR2. Increased ratios of G were detected in DDX6-KO HEK293T cells, demonstrating that DDX6 depletion indeed increased the editing activity of endogenous (myc-only) or overexpressed (myc-ADAR1 and myc-ADAR2) ADARs. We also examined the A-to-I editing levels of two endogenous genes, AZIN1 and GLI1, in WT and D6KO cells (Figure 3H). AZIN1 was reported as a target mainly edited via ADAR1, while GLI1 was the common target for both ADAR1 and ADAR2. The total RNA was extracted from both the WT and D6KO cells and then reverse-transcribed to generate cDNA. The A-to-I editing sites on the cDNA of these two genes were PCR-amplified, followed by Sanger sequencing. The A/G dual peaks of each gene were presented when the cells were transfected with myc-ADARs. We also observed increased G ratios at the editing sites of both the AZIN1 and GLI1 genes in D6KO cells. Thus, depletion of DDX6 increased ADAR activity, suggesting that DDX6 is a negative regulator of ADAR1 and ADAR2 in the nucleus.

### 2.4. The Helicase Activity of DDX6 Is Dispensable for Regulating ADARs

**Figure 4:**
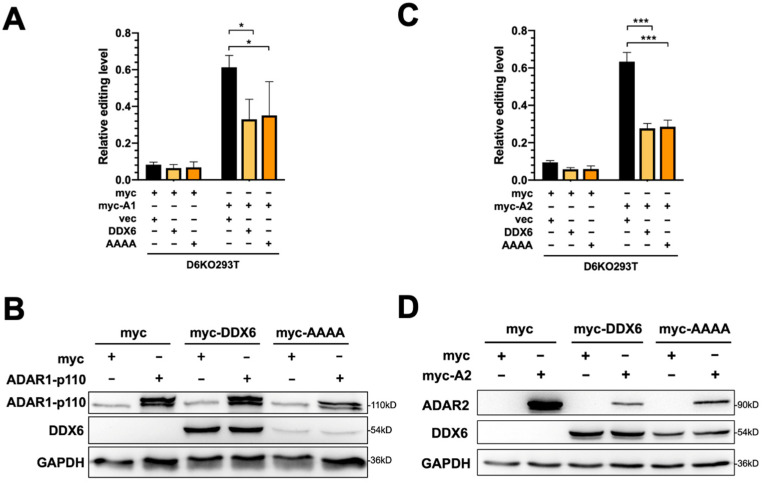
The helicase activity of DDX6 is dispensable for regulation of ADAR. (**A**) The A-to-I editing level of exogenous ADAR1p110 monitored with the DFR assay was reduced after expression of wild-type DDX6 (yellow) or its AAAA-mutant-lacking helicase activity (orange) in D6KO293T cells. Black, vector only controls. (**B**) WB analysis showed the equal amounts of exogenous myc-ADAR1 and expression of DDX6 constructs. (**C**) The A-to-I editing level of exogenous ADAR2 monitored with the DFR assay was reduced with expression of wild-type DDX6 (yellow) or its AAAA-mutant (orange) in D6KO293T cells. (**D**) WB analysis showed equal amounts of exogenous myc-ADAR2 and expression of DDX6 constructs. GAPDH, the loading control; myc-DDX6 or DDX6, wild-type DDX6; AAAA or myc-AAAA, the DDX6 mutant. The relative editing level was presented as normalization of the mCherry/GFP signal from pEGFP-Stop-P2A-mCherry to that from pEGFP-Stop-P2A-mCherry in cells that expressed myc-ADAR1 or myc-ADAR2. Statistical analysis was conducted using an unpaired, two-tailed Student’s *t* test (*N* = 3; *: *p* < 0.05; ***: *p* < 0.001).

To test whether restoration of DDX6 can rescue elevated ADAR activity in DDX6-KO cells, we transfected plasmid-expressing wild-type DDX6 in DDX6-KO HEK293T cells, then performed a DFR assay in cells transfected with ADAR1p110 or ADAR2. To test whether the helicase activity of DDX6 is involved in regulating ADARs, we also overexpressed helicase-null-mutant DDX6 (DEAD->AAAA) in D6KO cells, followed by the DFR assay (Figure 4A,C). The DDX6-wt and DDX6-AAAA mutant expressions were confirmed with Western blotting (Figure 4B,D). An increased ADAR-induced editing level in reporter mRNA was quantified as the fold change of mCherry/GFP in cells that expressed myc-ADAR1 (Figure 4A) or myc-ADAR2 (Figure 4C) to the mCherry/GFP signals observed in myc-only control. The effect of overexpression of DDX6 on repression of ADAR activity is presented as the relative fold change of editing levels compared to the vector control (Figure 4A,C, right panels). We found that overexpression of wild-type DDX6 reduced ADAR1-mediated editing to ~40% of the vector control (Figure 4A). A similar effect was observed in the DFR assay for ADAR2-mediated editing (Figure 4C). Interestingly, overexpression of DDX6-AAAA still significantly repressed ADAR1 and ADAR2 activity, suggesting that the DEAD-box helicase domain is dispensable for the DDX6 function in regulation of ADAR1 and ADAR2 in human cells.

### 2.5. The C-Terminal Domain of DDX6 Is Required for Regulation of ADARs

**Figure 5:**
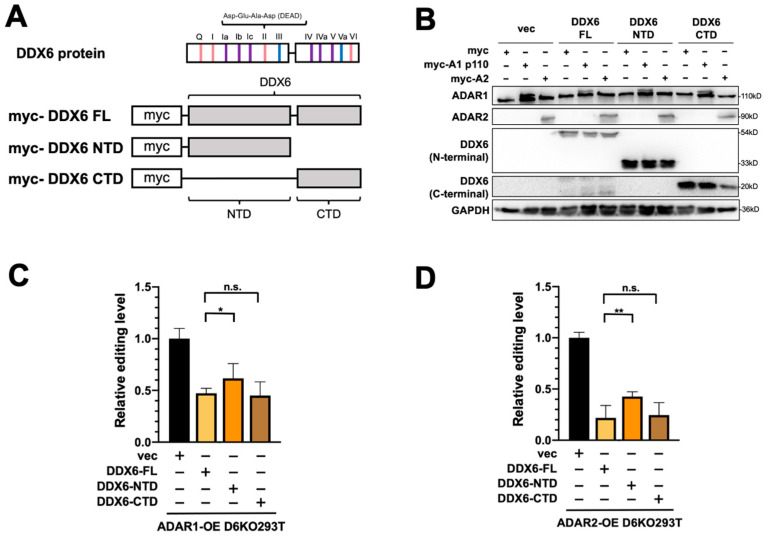
The C-terminal domain of DDX6 is required for regulation of ADAR editing activity. (**A**) This illustration shows the conserved motifs of the DDX6 protein and myc-tagged DDX6 constructs. Pink, ATP binding and hydrolysis; purple, RNA binding; blue, communication between ATP-binding and RNA-binding sites. The conserved amino acid sequence Asp-Glu-Ala-Asp (DEAD) was located on motif II (upper). NTD, the 1–300 AA of DDX6; CTD, the 300–483 AA of DDX6 (lower). (**B**) WB analysis showed expressions of exogenous myc-ADARs and various DDX6 constructs. GAPDH was the loading control. DDX6-FL and DDX6-NTD were detected with anti-DDX6 (A300-460A, Bethyl). DDX6-CTD was detected with anti-DDX6 (A300-461A, Bethyl). (**C**,**D**) DDX6-NTD was not sufficient to repress the A-to-I editing mediated with ADAR1 (**C**) or ADAR2 (**D**) to the same levels as what DDX6-FL or DDX6-CTD had in DDX6-KO HEK293T cells. The editing-level increase was measured in D6KO293T cells with DDX6-FL (yellow), DDX6-NTD (orange), or DDX6-CTD (brown). Relative editing levels were normalized to that of vec-control-transfected cells. Error bars indicate the standard deviation. Statistical analysis was conducted using an unpaired, two-tailed Student’s *t* test (N = 3; n.s.: no significance; *: *p* < 0.05; **: *p* < 0.01).

We previously showed that the C-terminal domain (CTD) of DDX6 facilitates the entry of DDX6 into the nucleus [37]. Since the interactions between DDX6 and ADARs were identified in the nuclear extracts, we speculated that the nuclear localization of DDX6 is also critical in regulating ADAR activity. To characterize the domain requirement of DDX6 in regulating ADAR activity, we performed the DFR assay in D6KO cells that overexpressed full-length DDX6 (DDX6-FL), the N-terminal domain of DDX6 (AA 1-300, DDX6-NTD), or the C-terminal domain (AA 300-483, DDX6-CTD) of DDX6 (Figure 5A). A-to-I editing mediated with ADAR1 or ADAR2 was monitored separately in D6KO293T cells that expressed wild-type (full-length), the NTD of, or the CTD of DDX6. The expressions of transfected DDX6 and ADARs were confirmed with Western blotting (Figure 5B). ADAR1 editing activity was repressed in a similar range when cells overexpressed DDX6-FL or DDX6-CTD (Figure 5C, ~45% and ~40% relative fold change to the vector control, respectively). However, the DDX6-NTD showed a mild repressive effect (~65% of the control), suggesting that DDX6-NTD is insufficient to repress ADARs. Our results indicated that the C-terminal domain, or the nuclear entry feature, of DDX6 is necessary for repression of ADAR activity.

### 2.6. DDX6 Is Upregulated in RA-Induced Differentiation of SH-SY5Y Neuroblastoma Cells

**Figure 6:**
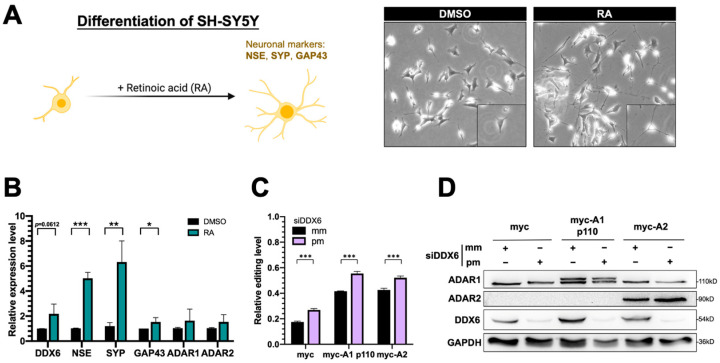
Expression of DDX6 was increased in SH-SY5Y cells that were treated with retinoic acid for differentiation. (**A**) The morphological changes in RA-induced differentiated SH-SY5Y cells. Cells treated with RA for 7 days showed long and branched neurites. Magnification 200×. (**B**) Quantitative analysis of mRNA levels of various genes, including the differentiation markers NSE, SYP, and GAP43, after RA-induced differentiation in SH-SY5Y cells. Expression of DDX6 was also increased when NSE and SYP were significantly upregulated with RA treatment. DMSO, solvent control for RA treatment; RA, retinoic acid. (**C**) The DFR assay showed the increased A-to-I editing level after knockdown DDX6 in SH-SY5Y cells. The relative editing level was presented as the mCherry/GFP signal from pEGFP-Stop-P2A-mCherry to that from pEGFP-Stop-P2A-mCherry in cells that expressed myc, myc-ADAR1, or myc-ADAR2. Error bars indicate standard deviation. Statistical analysis was conducted using an unpaired, two-tailed Student’s *t* test (*N* = 3; *: *p* < 0.05; **: *p* < 0.01; ***: *p* < 0.001). (**D**) Western blots confirmed depletion of DDX6 in SH-SY5Y cells that were transfected with siRNA that targeted DDX6 (siDDX6pm). pm, perfectly matched siRNA; mm, mismatched siRNA; GAPDH, the loading control.

The human SH-SY5Y neuroblastoma cell is considered a catecholaminergic neuron progenitor cell line and has been wildly used in the study of degenerative neuronal diseases. To examine the functions of DDX6 and the potentially antagonistic effect of ADARs in regulation of neuronal differentiation, we used SH-SY5Y as the model to test our hypothesis. Retinoic acid (RA) treatment induced differentiation of SH-SY5Y cells toward dopaminergic neurons with morphological changes (Figure 6A) and expression of neuronal marker genes, including NSE, SYP, and GAP43. With the RA treatment, we found that the expression of DDX6 was nearly twofold increased after differentiation, while ADAR1 and ADAR2 were not significantly changed (Figure 6B). These data suggest that the downstream pathways or biological processes facilitated via DDX6 could be simultaneously regulated with an increase in DDX6 level during differentiation. 

To test whether DDX6 regulates ADAR activity in SH-SY5Y cells and participates in differentiation, we performed the dual-fluorescence reporter assay in SH-SY5Y cells transfected with siRNA that was perfectly matched or mismatched to DDX6. Consistently with the results for HEK293T cells, depletion of DDX6 also significantly increased the A-to-I editing levels caused by ADAR1p110 and ADAR2 in SH-SY5Y cells (Figure 6C), suggesting that DDX6 also exhibits a repressive function in regulating ADAR activity in neuroblastoma cells and could be ubiquitous in various human cell types.

### 2.7. Both DDX6 and ADARs Participate in Neuronal Differentiation

**Figure 7:**
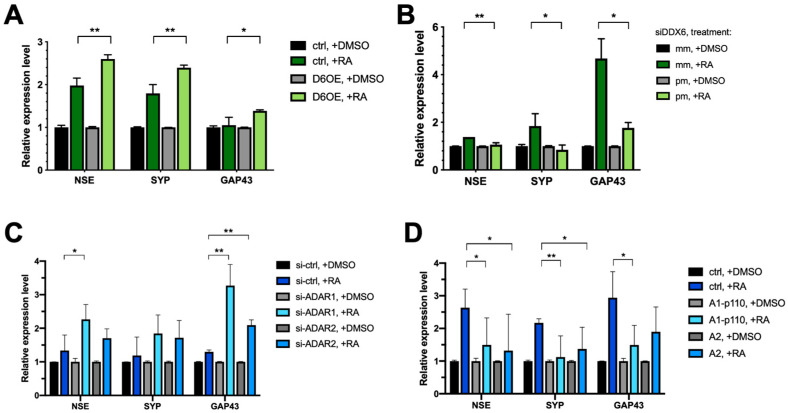
Both DDX6 and ADARs participate in differentiation of SH-SY5Y cells. (**A**) RT-qPCR analysis of neural differentiation markers NSE, SYP, and GAP43 in SH-SY5Y cells that overexpressed myc-DDX6. Expressions of differentiated markers were increased in DDX6-OE SH-SY5Y cells. (**B**) RT-qPCR analysis of the differentiation status in DDX6-depleted SH-SY5Y cells. Expressions of differentiation markers were decreased in DDX6-KD SH-SY5Y cells. (**C**) RT-qPCR analysis of neuron differentiation in SH-SY5Y cells transfected with either si-ADAR1 or si-ADAR2. The expression levels of NSE, SYP, and GAP43 were increased in the ADAR1-KD and ADAR2-KD SH-SY5Y cells. (**D**) RT-qPCR analysis of neuron differentiation in SH-SY5Y cells that overexpressed myc-ADAR1 or ADAR2. Expressions of differentiation markers were decreased in ADARs-OE SH-SY5Y cells. DMSO, solvent control for RA treatment; RA, retinoic acid; NSE, SYP, and GAP43, differentiated marker genes; Ctrl, empty vector (pcDNA3.1-myc); mm, mismatch; pm, perfect match. Error bars indicate standard deviation. Statistical analysis was conducted using an unpaired, two-tailed Student’s *t* test (*N* = 3; *: *p* < 0.05; **: *p* < 0.01).

To decipher the role of DDX6 in neuronal differentiation, we overexpressed myc-tagged, full-length DDX6 in SH-SY5Y cells. We tested whether increasing the DDX6 level would facilitate or repress RA-induced differentiation. The differential status was determined according to the expressions of three neuronal markers: NSE, SYP, and GAP43. Overexpression of DDX6 resulted in increased levels of all three marker genes of neuronal differentiation (Figure 7A). To further confirm the effect of DDX6 in regulation of SH-SY5Y differentiation, we knocked down DDX6 in SH-SY5Y cells, followed by RA induction, and then analyzed the expression levels of neuronal marker genes (Figure 7B). Depletion of DDX6 in cells transfected with perfectly matched siRNA (siDDX6 pm) was confirmed via RT-qPCR (Figure S2). In contrast to the results in cells with DDX6 overexpression, depletion of DDX6 significantly decreased the expressions of differentiation marker genes after RA treatment in SH-SY5Y cells (Figure 7B). These data indicate that DDX6 promotes RA-induced differentiation in SH-SY5Y cells.

The increased DDX6 level in RA-induced differentiated cells raised a possibility that the A-to-I editing level could be reduced without changing of the expressions of endogenous ADARs (Figure 6B), and reduced ADAR activity may facilitate differentiation of SH-SY5Y cells toward dopaminergic neurons. Thus, downregulation of ADAR expression might have similar effects in regulation of differentiation of SH-SY5Y cells, as we observed with DDX6 overexpression. To test this hypothesis, we knocked down ADAR1 and ADAR2 with siRNA (Figure S3A) and examined the expression levels of the neuronal marker genes after RA induction. As shown in Figure 7C, depletion of ADAR1 and ADAR2 significantly increased the expressions of NSE and GAP43 compared to in the cells transfected with the si-control. To further confirm the role of ADARs in regulation of differentiation, we overexpressed ADAR1p110 and ADAR2 in SH-SY5Y cells (Figure S3B), treated the cells with RA to induce differentiation, and then analyzed the expression levels of neuronal marker genes on day six post-RA-induction (Figure 7D).

In contrast to the results in ADAR-depleted cells, overexpression of ADAR1 or ADAR2 significantly decreased the levels of differential marker genes. These results suggest that DDX6 and ADARs exhibit antagonism influences in regulation of differentiation of SH-SY5Y cells. Similar results were observed when we tested the functions of DDX6 and ADARs in modulation of RA-induced differentiation of SK-N-SH cells (Figure S4). Our data indicated that DDX6 and ADARs participate in the neuronal differentiation process in RA-induced cell models. Moreover, the opposite effect of DDX6 and ADARs suggested that inhibitory regulation of ADAR activity via DDX6 may synergistically function in the same pathway.

## 3. Discussion

**Figure 8:**
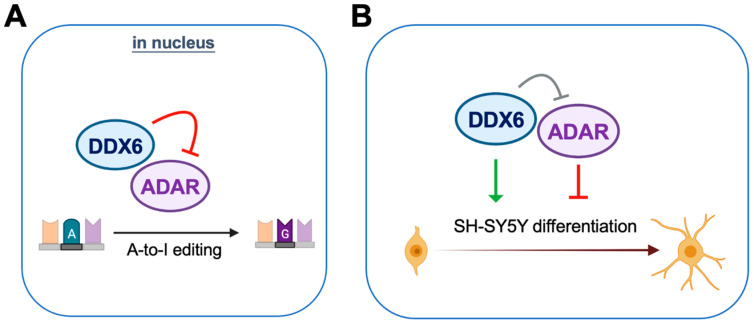
DDX6 negatively regulates ADAR editing activity and participates in neuronal differentiation. (**A**) DDX6 acts as a repressor of ADAR editing activity. (**B**) DDX6 promotes cell differentiation while ADARs inhibit the differentiation of the SH-SY5Y neuroblastoma cell.

DDX6 is ubiquitously expressed with conserved functions in regulation of RNA metabolism. In our mass-spec and immunoblotting analyses of DDX6-coimmunoprecipitated proteins, we found that ADAR1 and ADAR2 interact with nuclear DDX6. Since ADARs catalyze cellular A-to-I editing events, we hypothesize that DDX6 may participate in A-to-I RNA editing through regulation of ADAR activity. To further quantify the effect on RNA editing, we developed a dual-fluorescence reporter assay that contained a validated ADAR-editing site from Gria2 mRNA, which was previously applied in a luciferase reporter system for ADARs [40]. This reporter system was then employed to detect the A-to-I editing activity of ADARs via monitoring of the live fluorescence signal under microscopy or measuring it from cell lysate with a plate reader. With this DFR assay, we showed that nuclear DDX6 repressed A-to-I editing activity in both HEK293T cells and neuroblastoma progenitor SH-SY5Y cells. We also proved that, instead of the N-terminal domain, the C-terminal domain of DDX6 is sufficient for regulating ADARs, while the helicase activity of full-length DDX6 is dispensable. To examine the functions of DDX6 and ADARs in neuronal differentiation, we overexpressed or knocked down DDX6, as well as manipulating the ADAR1p110 and ADAR2 expression levels in the neuronal progenitor cell SH-SY5Y, followed by RA-induced differentiation. We found that overexpression of DDX6 or depletion of ADAR1 and ADAR2 increased the expression levels of differentiation markers in neuronal progenitor cells. Our results suggest that both DDX6 and ADARs participate in regulation of cell fate transition in the differentiation of the neuronal cell model (Figure 8). 

A-to-I RNA editing via ADARs is pervasive in metazoan [41,42]. In humans, three ADAR orthologs are characterized with conserved double-stranded RNA binding domains and deaminase domains, while only ADAR1 and ADAR2 are catalytically active. Expression of ADAR1 from alternative promoters results in two protein isoforms: p150 and p110. ADAR1p110 and ADAR2 are constitutively expressed mainly in the nucleus, whereas ADAR1p150 is expressed in cytoplasm and the nucleus upon interferon induction [43]. Consistently with previous reports, we barely observed the expression of ADAR1p150 in the nuclear fractions of the HeLa and HEK293T lysates and even in the total cell lysates. Thus, we mainly focused on the interactions of DDX6 with ADAR1p110 and ADAR2 in this study. Whether DDX6 interacts with and regulates IFN-induced ADAR1p150 in both the nucleus and the cytoplasm remains unknown and could be examinable in other cell types or conditions. 

The functions of human ADARs have been demonstrated in multiple aspects. ADAR1 mostly edits long, nearly perfect double-stranded regions of long transcripts from inverted repeats, such as Alu elements [44,45]. These types of editing are involved in self- and nonself-RNA recognition for innate immunity; thus, ADAR1 mutation causes immune-related disease [46,47,48]. Recent studies have also shown that the ADAR1 expression level and tumor aggressiveness are correlative, suggesting that ADAR1 is a potential target for cancer therapy [49,50,51]. Unlike ADAR1, ADAR2 majorly edits the adenosine in nonrepeated dsRNA in the brain [52]. Many editing events mediated with ADAR2 in the coding region of neuronal RNA are critical for protein functions after translation. For example, Gria2, which encodes a glutamine receptor and is used in our reporter constructs, is an ADAR2 target. In ADAR2 mutants, unedited Gria2 would express a calcium-permeable AMPA receptor, which would be harmful to the central nervous system [42]. Thus, temporal and spatial regulation of editing events is crucial for proper control of the expressions of target genes. Although target RNA sequence and structure play a critical role in determining editing levels, many studies have also shown that tissue-specific editing changes did not directly correlate with ADAR expression levels, suggesting that other protein factors may be complexed with ADARs in order to regulate editing events [52,53]. These ADAR transregulators, such as FMRP, DICER, DDX15, HNRNPA2/B1, TDP-43, Drosha, ELAVL1, and DHX9, have been reported to affect RNA editing [54,55]. However, only limited studies have shown the mechanism of how these interactors regulate ADARs. Among them, the role of DHX9 in ADARs has been well addressed. Aktaş et al. showed that DHX9 interacts with interferon-induced ADAR1p150 but not with constitutively expressed ADAR1p110 in the nucleus and suppresses Alu-dependent RNA processing defects through increased circular RNA generation [56]. In addition, another study showed that depletion of DHX9 decreased ADAR1-specific editing but increased ADAR2 editing, suggesting bidirectionally regulatory roles of DHX9 in A-to-I editing catalyzed with different ADARs [57]. This function of DHX9 is helicase-dependent, and dysfunction of or misregulated DHX9 reshapes the global editing profile and contributes to cancer development [57]. These data also suggest that one particular ADAR regulator could modulate RNA editing through various ADARs in different directions. 

Although DDX6 is present in the nucleus and the cytoplasm, the functions of DDX6 that have been elucidated are majorly in the cytoplasm. Here, we found that DDX6 interacts with ADAR1p110 and ADAR2 in the nuclei of HeLa and HEK293T cells. DDX6 has been mentioned in the lists of ADAR2-interacting proteins in previous DHX9 studies [57], but whether DDX6 participated in RNA editing had yet to be fully characterized. Through analysis of the RNA-seq datasets from two human cell lines generated upon depletion of various RBPs in the ENCODE project, Quinones-Valdez et al. found increased average editing levels at differentially edited sites in DDX6-depleted HepG2 cells but not in K562 cells, suggesting that DDX6 is a potential repressor of ADARs in specific cell types [55]. This evidence supports our finding that DDX6 is a negative regulator in RNA editing. Between this and our data, the function of DDX6 in RNA editing was observed or can be expected in at least four cell types. Whether this regulatory function is ubiquitous remains to be discovered. Thus, it will be interesting to survey the involvement of DDX6 in regulation of ADAR activity across different types of cells in order to clarify the tissue-specific roles of DDX6 in RNA editing. 

How does DDX6 regulate ADARs in the nucleus? The requirement of the helicase activity of DHX9 in regulation of ADARs raised the hypothesis that the functional significance of DDX6 on repression of RNA editing may be through a similar mechanism. The DEAD motif, the core motif of helicase activity, has been shown to participate in viral replication, P-body formation, and pluripotency exit in human iPSCs [31,58,59]. In our study, however, we showed that the DEAD-box motif is dispensable for the function of DDX6 in repression of ADARs. Thus, DDX6 may regulate ADARs via a mechanism different from what DHX9 has. In addition, DDX6-CTD could also repress ADARs, suggesting that the domains that dictate the function of DDX6 are located in the C-terminal region. Nuclear-localized DDX6-CTD contains one RecA-like domain and can still bind to RNA. It is possible that DDX6 inhibits ADARs via competition for dsRNA binding with ADARs or synergistic collaboration with other proteins, such as DHX9 or other cofactors. These potential mechanisms should be further decrypted with additional experiments in the future. 

The function of DDX6 in animal development has been addressed in several studies, while the role of DDX6 in neuronal differentiation remains elusive. A recent study showed that suppression of RNA helicase DDX6 in human and mouse embryonic stem cells (ESCs) results in a differentiation-resistant, "hyper-pluripotent" state through a P-body dissolution mechanism [31]. In addition, Kim et al. showed that DDX6 represses aberrant inhibition of BMP signals through miRNA-mediated pathways in control of early mouse embryogenesis [60]. Here, we delineated the potential roles of DDX6 in affecting differentiation of neuronal lineage cells and participating in ADAR-mediated RNA editing. To study the impact of DDX6 on neuronal cell differentiation, we used SK-N-SH and SH-SY5Y: two neuroblastoma cell lines. The SH-SY5Y cells, derived from SK-N-SH, had a neuroblast-like morphology with few and truncated processes. With specific treatments, SH-SY5Y cells can be differentiated to express catecholaminergic neurotransmitters or markers for cholinergic neurons. Therefore, SH-SY5Y cells are generally used as models for neurodegenerative disorders such as Parkinson’s disease and Alzheimer’s disease [61]. According to our results, overexpression of DDX6 promoted RA-induced differentiation of both SN-K-SH and SH-SY5Y cells. Interestingly, differentiation of SN-K-SH and SH-SY5Y cells upon RA treatment resulted in two different neuronal lineages: adrenergic and catecholaminergic. Thus, the impact of DDX6 in both lineages suggested that DDX6 may participate in the early stage of fate determination during neuronal differentiation. As the functions of ADAR1/ADAR2 in this process remain poorly understood, our findings also suggest a novel role of ADAR1/ADAR2 in regulating cell fate determination. Future work may explore whether DDX6 similarly affects other lineages, supporting our hypothesis. 

In sum, through identification of the protein associated with DDX6 in the nuclei of human cells, we uncovered that DDX6 interacts with ADAR1 and ADAR2. We have provided experimental evidence that DDX6 can regulate ADAR1/2-mediated RNA editing. We showed that the CTD of DDX6 is required for ADAR repression, while the DEAD helicase motif is dispensable. Moreover, DDX6 and ADARs are involved in RA-induced differentiation of neuronal cells, suggesting potential antagonistic impacts of DDX6 and ADARs in neuronal development. These findings have improved our understanding of the functional consequences of DDX6 in RNA processing and metabolism and provided an aspect of the novel roles of DDX6 in the nuclei of human cells.

## 4. Materials and Methods

### 4.1. Cell Culture

HEK293T cells, SH-SY5Y cells, and SK-N-SH cells were cultured at 37 °C, 5% CO_2_ in Gibco Dulbecco’s Modified Eagle Medium (DMEM) supplied with 10% fetal bovine serum (FBS) (Thermo Fisher Scientific, Carlsbad, CA, USA). 

### 4.2. Cell Transfection

Cells were seeded in 12-well plates and, after 24 h, subjected to transfection. Plasmids were transfected at 500 ng/well for gene overexpression, using Lipofectamine 3000 (Thermo Fisher Scientific, Carlsbad, CA, USA) for SH-SY5Y and SK-N-SH cells or Lipofectamine 2000 (Thermo Fisher Scientific, Carlsbad, CA, USA) for HEK293T cells, according to the manufacturer’s instructions. To knock down gene expressions, siRNA was transfected at 1 µL/well in the 12-well plate using Lipofectamine 3000 (Thermo Fisher Scientific, Carlsbad, CA, USA) according to the manufacturer’s instructions. After 24 h, for the dual-fluorescence reporter assay, ADARs were transfected at 300 ng/well and plasmids of reporters were transfected at 240 ng/well at the same time, using Lipofectamine 3000 (Thermo Fisher Scientific, Carlsbad, CA, USA) according to the manufacturer’s instructions. Empty vectors (pcDNA3.1-myc) were transfected to obtain an equal amount of DNA as a negative control. Cells were harvested for analysis after 24 h.

### 4.3. Cloning and Constructs

To express myc-DDX6, the coding region was PCR-amplified from pEYFP-DDX6 [37] and inserted into a pKR43 expression vector that contained a 1× N-terminal myc tag. Myc-tagged DDX6 mutants were generated from YFP-tagged DDX6 mutants via PCR amplification [37]. Myc-tagged AAAA, a helicase mutant of DDX6, was cloned via two-step PCR mutagenesis at the DEAD domain, from “GATGAGGCAGAT” to “GCTGCGGCAGCT”. Myc-tagged ADARs were cloned from PCR amplification of HEK293T cDNA into plasmid pcDNA3.1-myc using designed cloning primers (Supplementary Table S1). The reporter pEGFP-Stop-P2A-mCherry was cloned via insertion of ADAR2 editing sequencing [40] into pEGFP-P2A-mCherry. The mutated reporter, which mimicked 100% editing, was cloned using point mutation at the editing site, from UAG to UGG.

### 4.4. siRNA

For ADAR knockdown, we used siCtrl (siGENOME nontargeting siRNA pool, D-001206-13-05, GE/Dharmacon, Lafayette, CO, USA), siADAR1 (M-008630-01-0005, GE/Dharmacon, Lafayette, CO, USA), and siADAR2 (M-009263-01-0005, GE/Dharmacon, Lafayette, CO, USA). The siRNA sequences (passenger strands) against DDX6 were DDX6-pm: 5′-GCAGAAACCCUAUGAGAUUUU-3′ and DDX6-mm: 5′-GCAGAAACGGAAUGAGAUUUU-3′.

### 4.5. Total Cell, Cytoplasmic, and Nuclear Protein Extraction

Total cell extract (TCE) were prepared from HEK293T cells via extraction of the cells with an RIPA lysis buffer with 10% MPI (Sigma-Aldrich, St. Louis, MO, USA), then centrifugation at 12,000× *g* for 15 min. Cytoplasmic (Cyto) and nuclear (Nu) fractions were prepared using the Gagnon–Li method [62].

### 4.6. Immunoprecipitation

Protein lysates, prepared as described above, were precleared with Dynabeads (Thermo Fisher Scientific, Carlsbad, CA, USA) for 1 h on a 4 °C rotor. For anti-DDX6 IP or the control, IgG IP, the precleared lysates were incubated with anti-DDX6 antibodies (Bethyl, Montgomery, TX, USA, A300-460A) or purified-rabbit IgG (Bethyl, Montgomery, TX, USA, P120-101) separately, along with Dynabeads on a 4 °C rotor overnight. The beads were washed three times using an IP lysis buffer (0.2 mM EDTA; 20 mM HEPES, pH 7.9; 0.35% Triton X-10; 10 mM NaCl; 1 mM MgCl2) and another four times in an IP wash buffer (25 mM Tris-HCl, pH 8.0; 150 mM NaCl; 0.3% [*v*/*v*] Nonidet P-40). Afterward, the beads were boiled with a 1× sample buffer for 10 min, followed by SDS-PAGE and WB analysis.

### 4.7. CRISPR/Cas9 Gene Knockout

The DDX6-knockout HEK293T stable cell line was established via transfection, followed by puromycin selection. pX459-DDX6-sgRNA1 (sgRNA1: 5′-CAGCCAGGCGGCGACTTCGG-3′) and pX459-DDX6-sgRNA2 (sgRNA2: 5′-AGTGCCATTATTGATTGTGT-3′) were cotransfected into HEK293T cells with Lipofectamine 2000 (Thermo Fisher Scientific, Carlsbad, CA, USA) according to the manufacturer’s instructions. After 24 h, the culture medium was refreshed. The puromycin was added to eliminate cells without transfected plasmids. Then, the surviving single colonies were picked up for WB analysis to check the knockout effect. 

### 4.8. Dual-Fluorescence Reporter Assay (DFR)

To measure ADAR editing levels, cell lysates were collected using 100 μL of 1× RIPA lysis buffer with 10% MPI (Sigma-Aldrich, St. Louis, MO, USA), each well then centrifugating at 12,000× *g* for 15 min. The supernatants were collected. For each sample, 30 uL of supernatants were diluted with 300 μL of 1× RIPA lysis buffer and added, 100 μL per well, in triplicate in a 96-well microplate (Greiner bio-one, item No. 655076) for analysis. The well with only 100 μL of 1× RIPA lysis buffer added was used for detection of background signals. Fluorescence was detected with a SpectraMax i3x Multi-Mode Microplate Reader with SoftMax Pro Software 7.0.3 (Molecular Devices, San Jose, CA, USA). The excitation/emission wavelengths of GFP and mCherry were set as [485/510] nm and [585/610] nm. Signals were detected from the top as 10 flashes/read. The background signals were first deducted from the sample signals. Then, the ADAR editing level was calculated using the ratio of mCherry signals over GFP signals. Relative editing level was determined via normalization of the ratio from cells transfected with pEGFP-Stop-P2A-mCherry to its corresponding positive control, which was transfected with pEGFP-Trp-P2A-mCherry.

### 4.9. Western Blot Analysis

Protein lysates were extracted from cells via lysation with a RIPA lysis buffer with 10% MPI (Sigma-Aldrich, St. Louis, MO, USA), then centrifugation at 12,000× *g* for 15 min. Supernatants were collected and protein concentration was measured using a Bradford assay (Thermo Fisher Scientific, Carlsbad, CA, USA). Equal amounts of proteins were prepared to be separated with SDS-PAGE and transferred to PVDF membranes using the discontinuous (Tris/CAPS) buffer system (Bio-Rad, Hercules, CA, USA). The primary antibodies used were anti-ADAR1 (sc-73408, Santa Cruz Biotechnology, Dallas, TX, USA, 1:400), anti-ADAR2 (sc-73409, Santa Cruz Biotechnology, Dallas, TX, USA, 1:200), anti-DDX6 N-terminal (A300-460A, Bethyl, Montgomery, TX, USA, 1:5000), anti-DDX6 C-terminal (A300-461A, Bethyl, Montgomery, TX, USA, 1:5000), anti-GAPDH (GTX100118, GeneTex, Irvine, CA, USA, 1:10,000), anti-4E-T (A300-706A, Bethyl, Montgomery, TX, USA, 1:1000), and anti-Lamin a/c (ab108595, Abcam, 1:5000). The secondary antibodies were goat antirabbit IgG antibody-HRP (GTX213110-01, GeneTex, Irvine, CA, USA, 1:5000) and goat antimouse IgG-HRP (Santa Cruz Biotechnology, Dallas, TX, USA, 1:5000). Signals were developed using BM Chemiluminescence Blotting Substrate (11500694001, Roche, Switzerland). For Western blotting of immunoprecipitation, HRP-conjugated Trueblot antirabbit IgG secondary antibodies (18-8816-33, Rockland, Gilbertsville, PA, USA) were used.

### 4.10. RNA Extraction, Reverse Transcription, and Quantitative PCR (RT-qPCR)

Total RNA was extracted with TRIzol^®^ reagents. Reverse transcription was con-ducted using random hexamers (Thermo Fisher Scientific, Carlsbad, CA, USA) and SuperScript III (Thermo Fisher Scientific, Carlsbad, CA, USA). To quantify gene expression levels, equal amounts of cDNA were further analyzed with quantitative PCR using iQ SYBR Green Supermix (Bio-Rad, Hercules, CA, USA) with specific primer sets (Supplementary Table S2). The qPCR process started with denaturing for 3 min at 95 °C, followed by 44 cycles of 10 s at 95 °C, 20 s at 60 °C, and 10 s at 72 °C, then 10 s at 95 °C.

### 4.11. Target-Site PCR and Sanger Sequencing

The sequence of the target site was amplified via PCR using Phusion Polymerase (Thermo Fisher Scientific, Carlsbad, CA, USA) with different primer sets according to different genes (Supplementary Table S3). The amplified target fragments were purified with a QIAquick Gel Extraction Kit (QIAGEN), then sent for Sanger sequencing. The peak height ratio (G/A + G) defined the editing level.

### 4.12. Differentiation in Neuron Progenitor Cells

Cells were seeded in a 12-well plate for 24 h in Dulbecco’s Modified Eagle Medium (DMEM) supplied with 3% fetal bovine serum (FBS) (Thermo Fisher Scientific, Carlsbad, CA, USA). Twenty-four hours after transfection, the medium was refreshed, and then the SH-SY5Y and SK-N-SH cells were treated with 10 µM retinoic acid (R2625, Sigma-Aldrich, St. Louis, MO, USA) for the final concentration, using equal amounts of DMSO (RNBG1476, Sigma-Aldrich, St. Louis, MO, USA) as a control, for 4 and 6 days, respectively.

### 4.13. Mass Spectrometry and Analysis

The immunoprecipitated proteins were resolved with SDS-PAGE and visualized with silver staining (Silver Stain Plus Kit, Bio-Rad, 161-0449). Protein bands within specific MW ranges were excised from the gel and digested prior to LC-MS/MS analysis according to the service providers’ protocol [37].

### 4.14. Statistical Analysis

Quantitative data were presented as means ± SD (standard deviation). Statistical analyses were performed using GraphPad Prism 8 software. Statistical significance was assessed using an unpaired, two-tailed Student’s *t*-test. *p*-values of less than 0.05 were considered statistically significant. Details are indicated in the respective figure legends.

## Data Availability

The data presented in this study are available in this article and its Supplementary Materials.

## Supplementary Materials

The following supporting information can be downloaded at: https://www.mdpi.com/article/10.3390/ijms24043197/s1.
