# Supplementary material for: RNA Helicase DDX6 Regulates A-to-I Editing and Neuronal Differentiation in Human Cells
Source: Int J Mol Sci. 2023 Feb 6;24(4):3197. doi: 10.3390/ijms24043197 (PMC9965400; doi:10.3390/ijms24043197)
Supplement: Supplementary file 1 [file ijms-24-03197-s001.zip › ijms-2138559-supplementary.pdf]

Supplementary Materials:

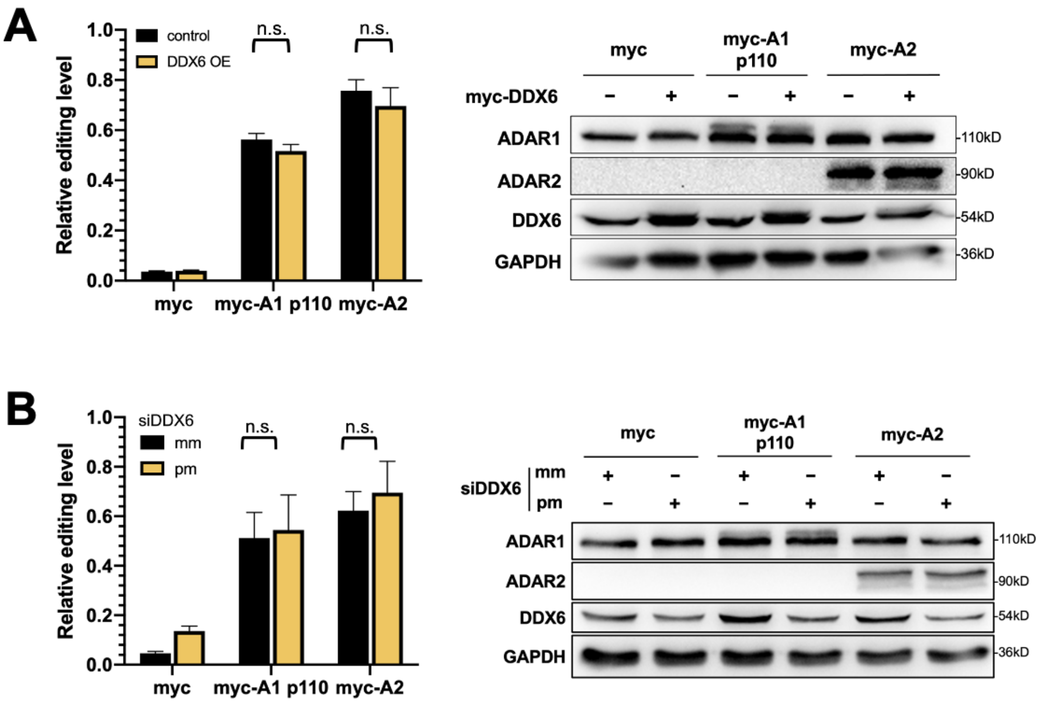

Figure S1. A-to-I editing level was changed in DDX6-OE or DDX6-KD HEK293T cells.

(**A**) ADAR editing level monitored by DFR assay in DDX6-OE HEK293T cells. The relative editing level was calculated by normalizing the mCherry/GFP ratio of pEGFP-Stop-P2A-mCherry to that in the pEGFP-Trp-P2A-mCherry group (left). WB analysis confirmed the overexpression of DDX6 in HEK293T cells (right). (**B**) The A-to-I editing level was monitored by DFR assay in DDX6-depleted HEK293T cells. The relative editing level was calculated by normalizing the mCherry/GFP ratio of pEGFP-Stop-P2A-mCherry to that in the pEGFP-Trp-P2A-mCherry group (left). WB analysis confirmed the efficiency of DDX6 RNAi in HEK293T cells (right). mm, mis-match. pm, perfect-match. GAPDH as the loading control. Error bars indicate standard deviation. Statistical analysis is conducted using un-paired, two-tailed Student's t test (*N*=3; n.s.: no significance).

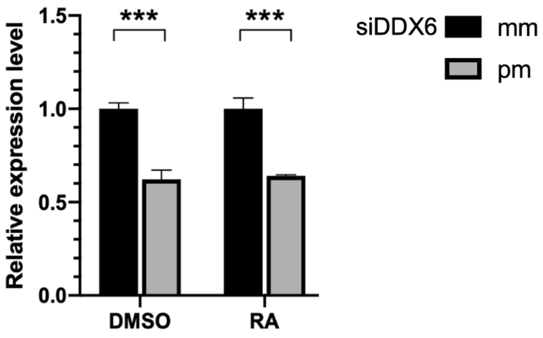

Figure S2. Depletion of DDX6 in cells transfected with siDDX6.
RT-qPCR analysis confirmed the RNAi effect of siDDX6 in SH-SY5Y cells. mm, mis-match control. pm, perfect-match. Error bars indicate standard deviation. Statistical analysis is conducted using un-paired, two-tailed Student’s t test (N=3; ***: $P<0.001$).

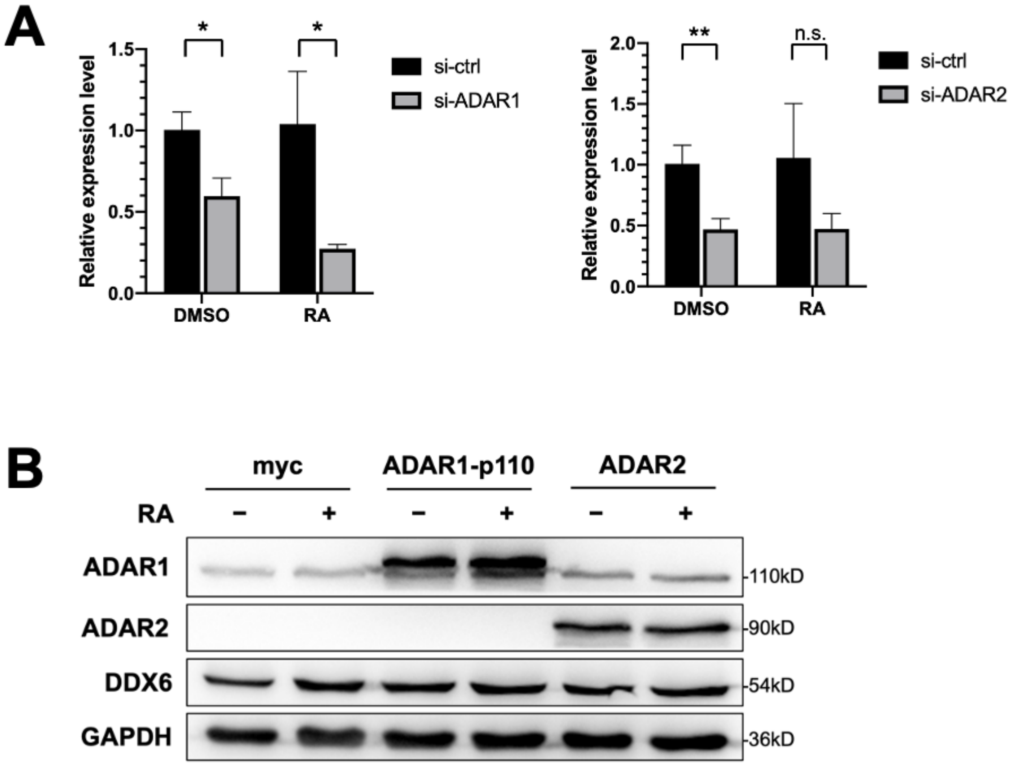

Figure S3. Depletion or overexpression of ADARs in SH-SY5Y cells.

(**A**) RT-qPCR analysis confirmed the efficiency of ADAR1 RNAi in SH-SY5Y cells (left). RT-qPCR analysis confirmed the efficiency of ADAR2 RNAi in SH-SY5Y cells (right). Error bars indicate standard deviation. Statistical analysis is conducted using un-paired, two-tailed Student's t test (*N*=3; n.s.: no significance; *: $P<0.05$; **: $P<0.01$). (**B**) WB analysis confirmed the overexpression of ADAR1p110 and ADAR2. GAPDH as the loading control. RA, retinoic acid.

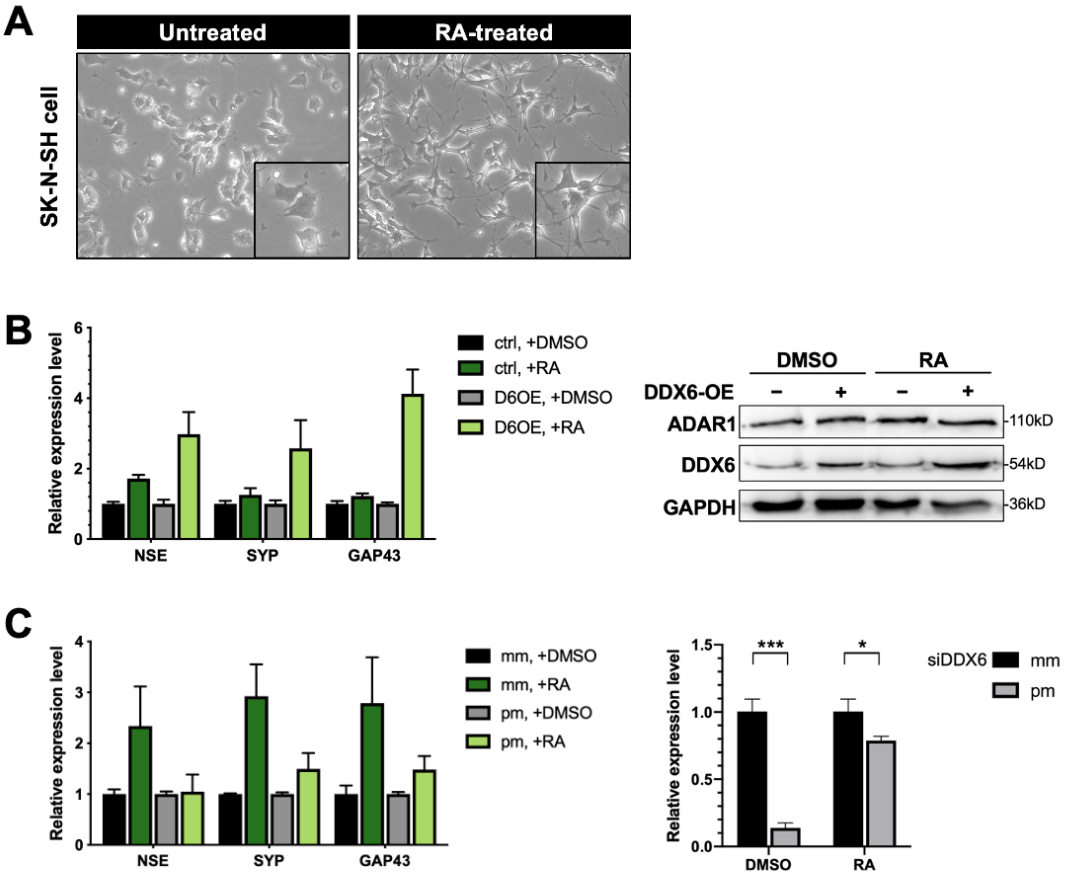

Figure S4. DDX6 promoted the differentiation of SK-N-SH cells.
(**A**) Morphological change in differentiated SK-N-SH cells. Cells exposed to RA became slender and angular. Magnification 200×. (**B**) RT-qPCR analysis of neuron differentiation in SK-N-SH cells overexpressing myc-DDX6. The differential status was monitored by the expression of marker genes, NSE, SYP and GAP43. The expression of marker genes was increased in DDX6-OE SK-N-SH cells (left). WB analysis confirmed the overexpression of DDX6 (right). (**C**) RT-qPCR analysis of neuron differentiation in DDX6-depleted SK-N-SH cells. The expression of marker genes was decreased in DDX6-KD SK-N-SH cells (left). RT-qPCR analysis confirmed the knockdown of DDX6 by siRNAs (right). GAPDH was the loading control. RA, retinoic acid. NSE, differentiated marker gene. SYP, differentiated marker gene. GAP43, differentiated marker gene. Ctrl, empty vector (pcDNA3.1-myc). mm, mis-match. pm, perfect-match. Error bars indicate standard deviation. Statistical analysis is conducted using un-paired, two-tailed Student's t test (*N*=3; *: P<0.05; ***: P<0.001).

Supplementary Table S1: List of ADAR cloning primers

	Primer sequence (5'→3')
ADAR1-p150_Fw	AAAAAAGCTTCATGAATCCGCGGCAGGGGT
ADAR1-p110_Fw	GGGGAAGCTTCATGGCCGAGATCAAGGAGAAAATC
ADAR1_Rv	GGTTGGGCCCCTATACTGGGCAGAGATAAAAGT
ADAR2_Fw	GGGGGAATTCTATGGATATAGAAGATGAAGAA
ADAR2_Rv	AAAACCGCGGTCAGGGCGTGAGTGAGAAC

Supplementary Table S2: List of qPCR primer sets

	Primer sequence (5’ → 3’)
GAPDH_Fw	CCATGAGAAGTATGACAACAGCC
GAPDH_RV	GGGTGCTAAGCAGTTGGTG
DDX6_Fw	GCTGGGAAAAGCCATCTCCTA
DDX6_Rv	GGTCTAGCCGTTCAAGTAAGGG
ADAR1_Fw	CATGATAGAGCAGAGTGGACC
ADAR1_Rv	CCTCTAGCAGAATTGTCATGGC
ADAR2_Fw	GGGTTTAGGCTGAAGGAGAAT
ADAR2_Rv	CTTGCTTTACGATTTGGGTGTC
NSE_Fw	CCCAGAACTTCCCTGATTGA
NSE_Rv	AAGTGGAAGACACGTGGGAC
SYP_Fw	TCTGGCCACCTACATCTTCC
SYP_Rv	TCAGCTCCTTGCATGTGTTC
GAP43_Fw	AGCCAAGCTGAAGAGAACATAG
GAP43_Rv	TCAGGCATGTTCTTGGTCAG

Supplementary Table S3: List of Target PCR primer sets and primers for sanger sequencing

gene	primers		Sequence (5'→3')
GRIA2	PCR	YFP-seq-Fw	GAGCAAAGACCCCAACGAG
		P2A-Rv	GCTCACCATAGGTCCAGGGTTCTCCTC
	sanger	P2A-Rv	GCTCACCATAGGTCCAGGGTTCTCCTC
AZIN1	PCR	AZIN1-seq-Fw	AATACAAGGAAGATGAGCCTCTGTTTAC
		AZIN1-seq-Rv	ACTGAATGACATCATGTAATAAATGGCT
	sanger	AZIN1-seq-Fw	AATACAAGGAAGATGAGCCTCTGTTTAC
GLI1	PCR	GLI1-seq-Fw	CAGCTACATCAACTCCGGCCAATAGGG
		GLI1-seq-Rv	AGCCTGGACCCCTCGCTCCATAAG
	sanger	GLI1-sanger	AGGCTGCTGACCGTCCTG

Supplementary Table S4: Mass Spectrometry data for nuclear DDX6-IP

Checked	Master	Accession	Gene	Description	Coverage	# Peptides	# PSMs	# Unique Peptides	# Protein Groups	# AAs	MW [kDa]	calc. pI	Found in Sample: 95 min run	Found in Sample: 50 min run	Protein FDR Confidence	Exp. q-value Mascot	Score Mascot	# Peptides Mascot
TRUE	Master Protein	P55265	ADAR	Double-stranded RNA-specific adenosine deaminase OS=Homo sapiens OX=9606 GN=ADAR PE=1 SV=4	2.446982	3	4	3	1	1226	136	8.65	High	High			72	3
TRUE	Master Protein	P07355	ANXA2	Annexin A2 OS=Homo sapiens OX=9606 GN=ANXA2 PE=1 SV=2	14.45428	4	10	4	1	339	38.6	7.75	High	High			220	4
TRUE	Master Protein	A6NMY6	ANXA2P2	Putative annexin A2-like protein OS=Homo sapiens OX=9606 GN=ANXA2P2 PE=5 SV=2	10.91445	3	5	3	1	339	38.6	6.95	High	High	High	0	128	3
TRUE	Master Protein	P02647	APOA1	Apolipoprotein A-I OS=Homo sapiens OX=9606 GN=APOA1 PE=1 SV=1	9.363296	2	3	2	1	267	30.8	5.76	High	High			112	2
TRUE	Master Protein	P06576	ATP5F1B	ATP synthase subunit beta, mitochondrial OS=Homo sapiens OX=9606 GN=ATP5F1B PE=1 SV=3	4.914934	2	2	2	1	529	56.5	5.4	High	Not Found			68	2
TRUE	Master Protein	Q99700	ATXN2	Ataxin-2 OS=Homo sapiens OX=9606 GN=ATXN2 PE=1 SV=2	3.274943	3	5	3	1	1313	140.2	9.57	High	High			122	3
TRUE	Master Protein	Q8WWM7	ATXN2L	Ataxin-2-like protein OS=Homo sapiens OX=9606 GN=ATXN2L PE=1 SV=2	3.72093	4	4	4	1	1075	113.3	8.59	High	High			62	4
TRUE	Master Protein	P01024	C3	Complement C3 OS=Homo sapiens OX=9606 GN=C3 PE=1 SV=2	2.88635	2	4	2	1	1663	187	6.4	High	Medium			153	2
TRUE	Master Protein	P0C0L4	C4A	Complement C4-A OS=Homo sapiens OX=9606 GN=C4A PE=1 SV=2	5.619266	4	5	4	1	1744	192.7	7.08	High	High			143	4
TRUE	Master Protein	P04040	Cat	Catalase OS=Homo sapiens OX=9606 GN=CAT PE=1 SV=3	7.020873	2	5	2	1	527	59.7	7.39	High	High	High	0	106	2
TRUE	Master Protein	Q9NPI6	DCP1A	mRNA-decapping enzyme 1A OS=Homo sapiens OX=9606 GN=DCP1A PE=1 SV=3	19.41581	6	9	6	1	582	63.2	6.25	High	High	High	0	277	6
TRUE	Master Protein	Q92841	DDX17	Probable ATP-dependent RNA helicase DDX17 OS=Homo sapiens OX=9606 GN=DDX17 PE=1 SV=2	9.876543	6	10	6	1	729	80.2	8.27	High	High	High	0	252	6
TRUE	Master Protein	O00571	DDX3X	ATP-dependent RNA helicase DDX3X OS=Homo sapiens OX=9606 GN=DDX3X PE=1 SV=3	20.54381	10	14	10	1	662	73.2	7.18	High	High	High	0	272	10
TRUE	Master Protein	Q7L014	DDX46	Probable ATP-dependent RNA helicase DDX46 OS=Homo sapiens OX=9606 GN=DDX46 PE=1 SV=2	2.327837	2	2	2	1	1031	117.3	9.29	Medium	Medium			31	2
TRUE	Master Protein	Q08211	DHX9	ATP-dependent RNA helicase A OS=Homo sapiens OX=9606 GN=DHX9 PE=1 SV=4	10.62992	10	20	10	1	1270	140.9	6.84	High	High			410	10
TRUE	Master Protein	Q02413	DSG1	Desmoglein-1 OS=Homo sapiens OX=9606 GN=DSG1 PE=1 SV=2	6.577693	5	8	5	1	1049	113.7	5.03	High	High	High	0	157	5
TRUE	Master Protein	P15924	DSP	Desmoplakin OS=Homo sapiens OX=9606 GN=DSP PE=1 SV=3	3.099965	8	12	8	1	2871	331.6	6.81	High	High	High	0	224	8
TRUE	Master Protein	Q6P2E9	EDC4	Enhancer of mRNA-decapping protein 4 OS=Homo sapiens OX=9606 GN=EDC4 PE=1 SV=1	2.783726	3	3	3	1	1401	151.6	5.86	Medium	High			78	3
TRUE	Master Protein	Q15029	EFTUD2	116 kDa U5 small nuclear ribonucleoprotein component OS=Homo sapiens OX=9606 GN=EFTUD2 PE=1 SV	5.144033	4	5	4	1	972	109.4	5	High	High			121	4
TRUE	Master Protein	P38919	EIF4A3	Eukaryotic initiation factor 4A-III OS=Homo sapiens OX=9606 GN=EIF4A3 PE=1 SV=4	6.082725	2	3	2	1	411	46.8	6.73	High	High	High	0	82	2
TRUE	Master Protein	Q5D862	FLG2	Filaggrin-2 OS=Homo sapiens OX=9606 GN=FLG2 PE=1 SV=1	1.045588	2	6	2	1	2391	247.9	8.31	High	High	High	0	88	2
TRUE	Master Protein	P21333	FLNA	Filamin-A OS=Homo sapiens OX=9606 GN=FLNA PE=1 SV=4	2.153381	4	4	4	1	2647	280.6	6.06	Medium	High			69	4
TRUE	Master Protein	Q96QA5	GSDMA	Gasdermin-A OS=Homo sapiens OX=9606 GN=GSDMA PE=1 SV=4	3.595506	2	2	2	1	445	49.3	5.29	Medium	Medium	High	0	33	2
TRUE	Master Protein	P04908	H2AC4	Histone H2A type 1-B/E OS=Homo sapiens OX=9606 GN=H2AC4 PE=1 SV=2	36.92308	2	5	2	1	130	14.1	11.05	High	High			106	2
TRUE	Master Protein	O60814	H2BC12	Histone H2B type 1-K OS=Homo sapiens OX=9606 GN=H2BC12 PE=1 SV=3	19.04762	2	5	2	1	126	13.9	10.32	High	High			100	2
TRUE	Master Protein	P07910	HNRNPC	Heterogeneous nuclear ribonucleoproteins C1/C2 OS=Homo sapiens OX=9606 GN=HNRNPC PE=1 SV=4	7.51634	2	4	2	1	306	33.7	5.08	Medium	High			84	2
TRUE	Master Protein	P52272	HNRNPM	Heterogeneous nuclear ribonucleoprotein M OS=Homo sapiens OX=9606 GN=HNRNPM PE=1 SV=3	28.90411	16	22	16	1	730	77.5	8.7	High	High	High	0	468	16
TRUE	Master Protein	Q00839	HNRNPU	Heterogeneous nuclear ribonucleoprotein U OS=Homo sapiens OX=9606 GN=HNRNPU PE=1 SV=6	10.78788	6	7	6	1	825	90.5	6	High	High			150	6
TRUE	Master Protein	P02790	HPX	Hemopexin OS=Homo sapiens OX=9606 GN=HPX PE=1 SV=2	16.66667	4	7	4	1	462	51.6	7.02	High	High			227	4
TRUE	Master Protein	Q86YZ3	HRNR	Hornerin OS=Homo sapiens OX=9606 GN=HRNR PE=1 SV=2	5.192982	4	6	4	1	2850	282.2	10.04	High	High			117	4
TRUE	Master Protein	Q12905	ILF2	Interleukin enhancer-binding factor 2 OS=Homo sapiens OX=9606 GN=ILF2 PE=1 SV=2	8.717949	2	6	2	1	390	43	5.26	High	High	High	0	205	2
TRUE	Master Protein	Q12906	ILF3	Interleukin enhancer-binding factor 3 OS=Homo sapiens OX=9606 GN=ILF3 PE=1 SV=3	3.131991	2	2	2	1	894	95.3	8.76	Not Found	High			64	2
TRUE	Master Protein	Q5T749	KPRP	Keratinocyte proline-rich protein OS=Homo sapiens OX=9606 GN=KPRP PE=1 SV=1	18.13472	8	13	8	1	579	64.1	8.27	High	High	High	0	161	8
TRUE	Master Protein	P02545	LMNA	Prelamin-A/C OS=Homo sapiens OX=9606 GN=LMNA PE=1 SV=1	3.463855	2	3	2	1	664	74.1	7.02	High	High	High	0	67	2
TRUE	Master Protein	P61626	LYZ	Lysozyme C OS=Homo sapiens OX=9606 GN=LYZ PE=1 SV=1	14.18919	2	3	2	1	148	16.5	9.16	High	Medium			65	2
TRUE	Master Protein	P43243	MATR3	Matrin-3 OS=Homo sapiens OX=9606 GN=MATR3 PE=1 SV=2	10.7438	7	12	7	1	847	94.6	6.25	High	High			245	7
TRUE	Master Protein	Q9HCE1	MOV10	Helicase MOV-10 OS=Homo sapiens OX=9606 GN=MOV10 PE=1 SV=2	2.691924	2	2	2	1	1003	113.6	8.82	Not Found	High			59	2
TRUE	Master Protein	P35749	MYH11	Myosin-11 OS=Homo sapiens OX=9606 GN=MYH11 PE=1 SV=3	4.107505	5	7	5	1	1972	227.2	5.5	High	High			189	5
TRUE	Master Protein	P06748	NPM1	Nucleophosmin OS=Homo sapiens OX=9606 GN=NPM1 PE=1 SV=2	12.92517	4	9	4	1	294	32.6	4.78	Medium	High			163	4
TRUE	Master Protein	Q5BJF6	ODF2	Outer dense fiber protein 2 OS=Homo sapiens OX=9606 GN=ODF2 PE=1 SV=1	4.82509	3	4	3	1	829	95.3	7.62	High	Medium			183	3
TRUE	Master Protein	P11940	PABPC1	Polyadenylate-binding protein 1 OS=Homo sapiens OX=9606 GN=PABPC1 PE=1 SV=2	24.5283	15	25	9	1	636	70.6	9.5	High	High	High	0	476	15
TRUE	Master Protein	Q13310	PABPC4	Polyadenylate-binding protein 4 OS=Homo sapiens OX=9606 GN=PABPC4 PE=1 SV=1	18.1677	11	19	5	1	644	70.7	9.26	High	High	High	0	409	11
TRUE	Master Protein	Q15366	PCBP2	Poly(rC)-binding protein 2 OS=Homo sapiens OX=9606 GN=PCBP2 PE=1 SV=1	6.027397	2	4	2	1	365	38.6	6.79	Medium	High			74	2
TRUE	Master Protein	Q6S8J3	POTEE	POTE ankyrin domain family member E OS=Homo sapiens OX=9606 GN=POTEE PE=2 SV=3	3.627907	2	4	1	1	1075	121.3	6.2	High	High			157	2
TRUE	Master Protein	Q9UKM9	RALY	RNA-binding protein Raly OS=Homo sapiens OX=9606 GN=RALY PE=1 SV=1	7.189542	2	2	2	1	306	32.4	9.17	Medium	Not Found			48	2
TRUE	Master Protein	Q96PK6	RBM14	RNA-binding protein 14 OS=Homo sapiens OX=9606 GN=RBM14 PE=1 SV=2	6.875934	4	6	4	1	669	69.4	9.67	Medium	High	High	0	86	4
TRUE	Master Protein	P38159	RBMX	RNA-binding motif protein, X chromosome OS=Homo sapiens OX=9606 GN=RBMX PE=1 SV=3	17.13555	7	13	7	1	391	42.3	10.05	High	High	High	0	281	7
TRUE	Master Protein	P05388	RPLP0	60S acidic ribosomal protein P0 OS=Homo sapiens OX=9606 GN=RPLP0 PE=1 SV=1	21.13565	6	9	6	1	317	34.3	5.97	High	High			255	6
TRUE	Master Protein	P08865	RPSA	40S ribosomal protein SA OS=Homo sapiens OX=9606 GN=RPSA PE=1 SV=4	28.47458	5	12	5	1	295	32.8	4.87	High	High	High	0	380	5
TRUE	Master Protein	Q92804	TAF15	TATA-binding protein-associated factor 2N OS=Homo sapiens OX=9606 GN=TAF15 PE=1 SV=1	15.87838	4	10	4	1	592	61.8	8.02	High	High	High	0	211	4
TRUE	Master Protein	Q9Y4P3	TBL2	Transducin beta-like protein 2 OS=Homo sapiens OX=9606 GN=TBL2 PE=1 SV=1	5.592841	2	5	2	1	447	49.8	9.44	High	High	High	0	168	2
TRUE	Master Protein	Q08188	TGM3	Protein-glutamine gamma-glutamyltransferase E OS=Homo sapiens OX=9606 GN=TGM3 PE=1 SV=4	3.896104	2	2	2	1	693	76.6	5.86	Medium	High	High	0	74	2
TRUE	Master Protein	P68363	TUBA1B	Tubulin alpha-1B chain OS=Homo sapiens OX=9606 GN=TUBA1B PE=1 SV=1	9.312639	2	3	2	1	451	50.1	5.06	Medium	High			59	2
TRUE	Master Protein	P04350	TUBB4A	Tubulin beta-4A chain OS=Homo sapiens OX=9606 GN=TUBB4A PE=1 SV=2	6.981982	2	2	1	1	444	49.6	4.88	Not Found	High			83	2
TRUE	Master Protein	P68371	TUBB4B	Tubulin beta-4B chain OS=Homo sapiens OX=9606 GN=TUBB4B PE=1 SV=1	6.966292	2	3	1	1	445	49.8	4.89	Not Found	High			173	2
TRUE	Master Protein	Q01081; P(	U2AF1	Splicing factor U2AF 35 kDa subunit OS=Homo sapiens OX=9606 GN=U2AF1 PE=1 SV=3; Splicing factor U2	14.58333	2	3	2	1	240	27.9	8.81	Medium	Medium			50	2
TRUE	Master Protein	P22695	UQCRC2	Cytochrome b-c1 complex subunit 2, mitochondrial OS=Homo sapiens OX=9606 GN=UQCRC2 PE=1 SV=3	6.401766	2	5	2	1	453	48.4	8.63	High	High	High	0	127	2
TRUE	Master Protein	Q5T750	XP32	Skin-specific protein 32 OS=Homo sapiens OX=9606 GN=XP32 PE=1 SV=1	8.8	2	3	2	1	250	26.2	7.97	Medium	High	High	0	52	2
TRUE	Master Protein	Q5BKZ1	ZNF326	DBIRD complex subunit ZNF326 OS=Homo sapiens OX=9606 GN=ZNF326 PE=1 SV=2	7.044674	3	3	3	1	582	65.6	5.15	High	Medium	High	0	67	3
